# The Effects of Preoperative Volume Replacement in Diabetic Patients Undergoing Coronary Artery Bypass Grafting Surgery: Protocol for a Randomized Controlled Trial (VeRDiCT Trial)

**DOI:** 10.2196/resprot.7386

**Published:** 2017-06-19

**Authors:** Madeleine Clout, Tracy Harris, Chris Rogers, Lucy Culliford, Jodi Taylor, Gianni Angelini, Pradeep Narayan, Barnaby Reeves, James Hillier, Kate Ashton, Kunal Sarkar, Raimondo Ascione

**Affiliations:** ^1^ Bristol Cardiovascular Faculty of Health Science University of Bristol Bristol United Kingdom; ^2^ Rabindranath Tagore International Institute of Cardiac Sciences Kolkata India

**Keywords:** coronary artery bypass surgery, diabetes mellitus, renal failure, volume replacement therapy, clinical trials, randomized

## Abstract

**Background:**

Diabetes mellitus is a major risk factor for prolonged hospital stays, renal failure, and mortality in patients having coronary artery bypass grafting (CABG). Complications pose a serious threat to patients and prolong intensive care and hospital stays. Low glomerular filtration rate (GFR) due to existing renal impairment or volume depletion may exacerbate acute renal impairment/failure in these patients. Preoperative volume replacement therapy (VRT) is reported to increase the GFR and we hypothesize that VRT will reduce renal impairment and related complications in diabetic patients.

**Objective:**

The objective of this study is to establish the efficacy of preoperative VRT in reducing postoperative complications in diabetic patients undergoing CABG surgery. Time to “fit for discharge”, incidence of postoperative renal failure, cardiac injury, inflammation, and other health outcomes will be investigated.

**Methods:**

In this open parallel group randomized controlled trial, 170 diabetic patients undergoing elective or urgent CABG surgery received 1 mL/kg/hour of Hartmann’s solution for 12 consecutive hours prior to surgery, versus routine care. The primary outcome was time until participants were “fit for discharge”, which is defined as presence of: normal temperature, pulse, and respiration; normal oxygen saturation on air; normal bowel function; and physical mobility. Secondary outcomes included: incidence of renal failure; markers of renal function, inflammation, and cardiac damage; operative morbidity; intensive care stay; patient-assessed outcome, including the Coronary Revascularization Outcome Questionnaire; and use of hospital resources.

**Results:**

Recruitment started in July 2010. Enrolment for the study was completed in July 2014. Data analysis commenced in December 2016. Study results will be submitted for publication in the summer of 2017.

**Conclusions:**

VRT is a relatively easy treatment to administer in patients undergoing surgical procedures who are at risk of renal failure. This experimental protocol will increase scientific and clinical knowledge of VRT in diabetic patients undergoing elective or urgent CABG surgery. Findings supporting the efficacy of this intervention could easily be implemented in the health care system.

**Trial Registration:**

International Standard Randomized Controlled Trial Number (ISRCTN): 02159606; http://www.controlled-trials.com/ISRCTN02159606 (Archived by WebCite at http://www.webcitation.org/6rDkSSkkK)

## Introduction

Diabetes mellitus (DM) has been recognized as a major risk factor for atherosclerosis [1,2], and its prevalence is on the rise due to an increasingly aging and obese population [3]. DM is also a major risk factor for postoperative renal failure, infections, and in-hospital and late mortality in patients undergoing coronary artery bypass grafting (CABG) surgery [4,5]. Diabetic patients currently represent approximately 20% of all patients undergoing CABG surgery [3,4,6-8]. This rate does not include undiagnosed DM, which has been reported to be 5.2% of all patients admitted for CABG [6]. In keeping with the United Kingdom (UK) average, approximately 20% of CABG patients have a diagnosis of DM on admission at our institution [4]. However, we have previously demonstrated that only 40% of patients experiencing postoperative moderate-to-poor blood sugar control after CABG were diagnosed as having DM prior to surgery [9]. This finding suggests that the prevalence of undiagnosed DM in patients admitted for CABG may be grossly underestimated, and emphasizes the importance of optimizing the perioperative management of these patients.

Renal insufficiency and acute kidney injury (AKI) remain frequent and serious complications following cardiac surgery, and are associated with other postoperative complications, mortality, prolonged hospital stays, and costs [7-9]. AKI following CABG is also consistently associated with DM [7-9]. Our group has demonstrated that DM is an independent predictor of renal insufficiency in a large cohort study [9], consistent with previous reports linking DM with AKI [8]. AKI can be defined in various ways. The introduction of the Risk, Injury, Failure, Loss, and End stage (RIFLE) criteria have standardized the National Health Service (NHS) definition of AKI and allowed more objective comparisons between different studies [10]. In its most severe form, AKI requires dialysis [10-12]. Death is 7-8 times more frequent in patients requiring dialysis because of AKI [13], but less severe renal dysfunction is also strongly associated with mortality [14]. AKI is reported to be more common after CABG in diabetic patients compared to nondiabetic patients [6,9].

Body fluid volume depletion may also be prevalent in patients undergoing CABG, especially diabetics. One factor leading to volume depletion is preoperative use of diuretics, which can cause hypovolemia, which reduces cardiac preload, cardiac output, and related organ perfusion. In addition, the use of vasodilators can also lead to relative volume depletion and hypotension [15]. Diminished renal perfusion can be a consequence of volume depletion, and of hemodynamic changes associated hypovolemia or with impaired left ventricular function [15,16]. In addition, agents such as nonsteroidal antiinflammatory drugs, cyclosporine, angiotensin-converting enzyme inhibitors, and angiotensin II receptor blockers can decrease renal perfusion [17-19]. In these situations, the resultant diminution in renal blood flow and estimated glomerular filtration rate (eGFR) can lead to postoperative renal insufficiency, and surgical patients could benefit from mild preoperative fluid replacement therapy since this has been associated with an increase in GFR [20]. A reduced eGFR may simply be a reflection of ongoing baseline renal impairment, which is also typical of diabetic patients.

Isotonic crystalloid solutions, such as Hartmann’s solution, are the first choice for volume replacement therapy (VRT) [21]. Unlike plasma expanders, crystalloid solutions have no nephrotoxic (or other specific) side-effects [21]. Isotonic crystalloid solutions are distributed rapidly into the tissue interstitial compartment and have a half-life of 20-30 minutes in the intravascular space.

This study protocol seeks to examine the effects of preoperative gentle VRT on postoperative time to “fitness for discharge”, renal failure, inflammation, cardiac injury, postoperative complications, and mortality in diabetic patients undergoing CABG surgery. A definitive trial of VRT in this indication is required before this relatively easy-to-administer treatment can be proposed as adjuvant therapy in diabetic patients undergoing cardiac surgery, or indeed other major surgical procedures. We hypothesize that the postoperative incidence of renal failure will be lower, and postoperative recovery faster, in diabetic patients treated with gentle VRT prior to surgery.

The main objective of the Volume Replacement in Diabetic Patients Undergoing Coronary Artery Bypass Grafting Surgery: Protocol for a Randomized Controlled Trial (VeRDiCT) is to evaluate the effect of VRT versus routine care on time until participants are “fit for discharge”, which is defined as normal temperature, pulse, respiratory rate, oxygen saturation on air, bowel function, and physical mobility. Secondary objectives will be to evaluate the effect of VRT versus routine care on the incidence of: postoperative renal failure and biochemical serial markers of renal function, inflammation, and cardiac damage; operative morbidity; intensive care stay; and use of hospital resources. In addition, we will evaluate patient-assessed outcome, which will be based on the serial administration of the Coronary Revascularization Outcome Questionnaire (CROQ).

We are conducting an open, parallel group, randomized controlled trial (RCT) in which diabetic elective or urgent patients undergoing CABG surgery will receive VRT or routine care. This treatment has already been shown to prevent AKI in certain clinical scenarios [21]. The most likely mechanism of action of VRT is by increasing eGFR [20]. It has also been suggested that VRT is essential to obtain adequate systemic circulation and microcirculation [22].

## Methods

### Type of Clinical Trial

This is an open, parallel group, RCT of preoperative VRT with Hartmann’s solution versus routine care in diabetic patients undergoing CABG. The trial is not blinded, as it is not possible to mask the infusion of the Hartmann’s solution. A covered drip could have been set up for all trial patients but it would still be obvious to the patient and those responsible for their care whether or not a fluid infusion was being given.

### Study Setting

This study was conducted in Bristol, UK at the Bristol Heart Institute, University Hospital Bristol NHS Foundation Trust. A parallel study was run at Rabindranath Tagore International Institute of Cardiac Sciences (RTIICS), Kolkata, India. The results of both trials are being combined and recruitment at both sites has contributed to the target sample size. RTIICS was responsible for research governance and approvals of their study.

### Ethical Review

The University Hospital Bristol NHS Foundation Trust has sponsored the trial in the UK. The trial was approved by the North Somerset & South Bristol Research Ethics Committee (REC; reference 10/H0106/1) in February 2010 in the UK. The study is a Clinical Trial of an Investigational Medicinal Product (IMP) and was approved by the UK Medicines and Healthcare Products Regulatory Agency (MHRA) in 2010. The study is registered (ISRCTN 02159606).

### Participants

**Figure 1:**
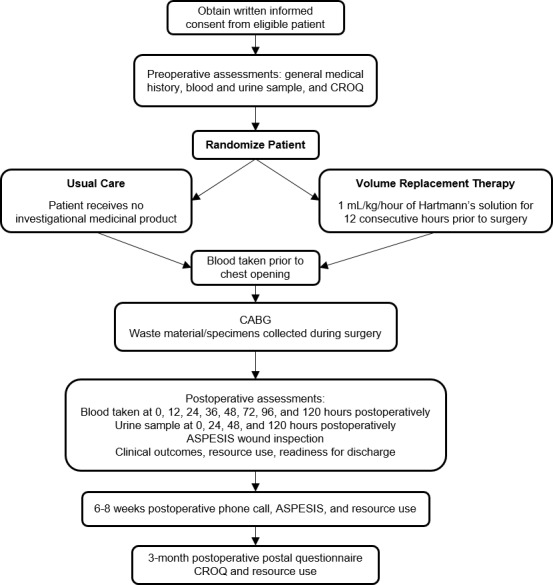
Study flow chart.

For this study, 170 diabetic patients undergoing CABG surgery were recruited to the two parallel studies according to the flow chart shown in Figure 1.

### Participant Recruitment

To assess eligibility, a member of the local research team (study clinician/research nurse/trial coordinator) in collaboration with the chief investigator (CI) has assessed patients’ medical notes to assess eligibility. VRT can be used in most adult diabetic patients for whom CABG is planned. Therefore, all diabetic adults aged >16 years and <80 years having elective or urgent isolated CABG for the first time represented the target study population. Participants could enter the study if all of the following applied: (1) patient was diagnosed with type I or type II diabetes, treated with oral medication and/or insulin (ie, not diet controlled only); (2) aged >16 and <80 years; (3) underwent elective or urgent isolated first-time CABG; (4) left ventricular ejection fraction >30%. Participants were excluded from the study if they had: (1) undergone previous cardiac surgery; (2) emergency or salvage operation; (3) chronic renal failure requiring dialysis; (4) current congestive heart failure; (5) left ventricular ejection fraction <30%.

### Information and Consent

Potential trial participants were identified from CABG waiting lists (elective patients) and theatre schedules (urgent patients). All potential participants were sent or given an invitation letter and Patient Information Sheet (PIS) approved by the REC, which described the study. The patient was given time to read the PIS and to discuss their participation with others outside the research team (eg, relatives or friends) if they wished. Most patients had at least 24 hours to consider whether they would participate. In a few cases this time interval was shorter (eg, for patients admitted for urgent surgery without prior notification to the waiting list coordinator). Details of all patients approached for the trial and reason(s) for nonparticipation (eg, reason for being ineligible or patient refusal) were documented. Signed informed consent was required and taken from all eligible patients who were willing to participate.

### Study Medication

Hartmann’s solution is classed as an IMP and is therefore under the regulation of the MHRA. The IMP was only administered to patients randomized to the VRT arm (there was no placebo). The dosage of the solution is dependent on body mass as follows: 1mL/kg/hour for 12 hours; therefore, maximum dose (mL) = body mass x 12.

Study medication was stored at room temperature in a temperature-monitored lockable cupboard. If the surgery was delayed and rescheduled to take place the next calendar day (morning or afternoon slot), the intervention was not to be repeated. If the surgery was delayed for 2 days or more, the intervention was repeated, and the IMP was represcribed.

### Procedure

Patients were randomly assigned in a 1:1 ratio using an Internet-based randomization system (Sealed Envelope Ltd). Cohort minimization was used to achieve balance between groups. Random allocations were generated by computer once the relevant baseline data required to identify the patient and establish eligibility were entered into the system. Consented patients were randomized in the evening on the day of admission, with the VRT intervention delivered overnight prior to surgery. If the operation was unexpectedly rescheduled, the patient retained their study number and randomized allocation. There was no requirement to have code breaking procedures in place. The trial was unblinded and the treatment was recorded in the medical notes and on the fluid chart.

Participants were randomly assigned to receive either VRT (1 mL/kg/hour of Hartmann’s solution for 12 consecutive hours prior to surgery) or usual care (no additional preoperative fluids). Both groups were fasted for 6 hours prior to surgery. At surgery, patients were managed according to routine anesthetic, surgical, and perfusion standards. Undertaking of coronary surgery with or without cardiopulmonary bypass and cardioplegic arrest were performed according to the surgeon’s preference. All other aspects of the patient’s preoperative and postoperative management were in accordance with existing protocols in use. The VRT was the only intervention administered over and above the usual care. Participants were asked to donate small blood and urine samples before, during, and after surgery, as well as any leftover tissue available during surgery that would normally be discarded. At discharge, participants were asked to indicate how they felt about their readiness to go home. At 6-8 weeks postsurgery, participants were asked to complete a telephone assessment of their wounds to ascertain presence of infection and answer a question regarding readmissions. Wounds were assessed using a recognized quantitative scoring method that provides a numerical score related to the severity of wound infection using objective criteria, including additional treatment, serous discharge, erythema, purulent exudate, separation of the deep tissues, isolation of bacteria, and the duration of inpatient stay (ASEPSIS). Three months after surgery, participants were asked to complete the CROQ. Key data collection points are shown in Table 1.

**Table 1:** Key data collection points of measured outcomes.

	Pre-surgery	Day of surgery	Day 1	Day 2	Day 3	Day 4	Day 5	Discharge	6-8 weeks	3 months
Eligibility	✓									
Written consent	✓									
Randomized allocation	✓									
Demographics and past medical history	✓									
Blood for serum creatinine (AKI/ eGFR) and other biochemical predictors of health outcome	✓	✓ ✓ (0, 12 hours)	✓ ✓ (24, 36 hours)	✓	✓	✓	✓			
Operative details		✓								
Clinical outcomes								✓		
CROQ	✓									✓
Readiness for discharge								✓		
ASEPSIS wound inspection					✓		✓	✓		
ASEPSIS post-discharge surveillance									✓	
Resource use data								✓	✓	✓
Urine sample (renal glomerular and tubular injury, microRNA and other biochemical predictors of health outcome)	✓	✓	✓	✓			✓			
Blood for fasting glucose and hemoglobin A1c	✓ (prior to chest opening)									
Blood for serum and plasma microRNA	✓	✓	✓				✓			
Leftover material/specimens collected during surgery		✓ (intra-operatively)								
Blood for Troponin T	✓	✓ ✓ (0, 12 hours)	✓	✓	✓		✓			
Blood for C-reactive protein	✓	✓ ✓ (0, 12 hours)	✓	✓	✓		✓			

## Results

### Primary Outcome

The primary outcome is the time until patients are classified as “fit for discharge”, since prevention of renal impairment by the proposed intervention is expected to impact the risk of many postoperative complications. A patient must have normal temperature, pulse, respiratory rate, oxygen saturation on air, and bowel function and be physically mobile (taking into account preoperative mobility such as wheel chair use) in order to be classified as “fit for discharge”.

### Secondary Outcomes

Secondary outcome measures include: (1) measurements of serum creatinine from blood samples collected preoperatively and postoperatively; (2) microalbumin/creatinine ratio measured in urine samples collected preoperatively and postoperatively; (3) N-acetyl-beta-D-glucosaminidase release measured in urine samples collected preoperatively and postoperatively; (4) participants’ judgement about readiness for discharge; (5) in-hospital mortality and morbidity; (6) use of health care resources; (7) health-related quality of life as measured by the CROQ; (8) preoperative fasting blood glucose; (9) micro-ribonucleic acid (RNA) measured in preoperative and postoperative urine, serum, and plasma samples; (10) C-reactive protein (CRP) measured preoperatively and postoperatively; and (11) serial troponin T release measured preoperatively and postoperatively. Measures 8-11 were only included in a subgroup of the UK trial.

### Safety Reporting

#### Side Effects

No previous randomized trials of VRT during cardiac surgery have been carried out, or were ongoing, at the time of the study. Hartmann’s solution is widely used for VRT, and is not known to cause allergic reactions or hemodynamic instability in this patient group. In this study, the solution was given at a very slow rate to have minimal impact on the well-being of the patient preoperatively. The solution was administered via a peripheral line that the patient would always have inserted at some point during their admission, so this was not considered to pose any additional risk.

#### Withdrawal of Individual Participants

Participants could withdraw from the study at any time for any reason and without any sanction. Researchers, after consulting with the CI and the study coordinator, could also interrupt the treatment program if, in their opinion, continuing the treatment may affect the patient’s welfare.

#### Suspension of the Study

In cases of suspected severe adverse events related to the administration of the treatment, the study could be interrupted and the researchers and coordinator would decide whether to continue.

#### Reporting of Adverse Events

Adverse events were recorded from the randomization time point, throughout the duration of the participant’s postoperative hospital stay, and for the predefined 3-month follow-up period. Any adverse event spontaneously reported by the participant, or observed by the researcher or the research team, was be recorded on the case report form (CRF) that was designed for this purpose. Events were also reported in accordance with the International Conference for Harmonization of Good Clinical Practice guidelines and followed the sponsor’s policy for safety reporting. Expected events included those related to administering Hartmann’s solution, as well as complications associated with cardiac surgery.

#### Anticipated Benefits

Potential benefits to participants include the possibility of improved renal protection for the intervention group, which we hypothesize will lead to a reduced incidence of postoperative renal insufficiency and a faster postoperative recovery. Should our hypothesis be supported by the findings of the trial, all future diabetic patients undergoing CABG should benefit from preoperative volume replacement. The main benefit to society is the provision of high quality evidence to address this important area of clinical uncertainty.

### Data Analysis

#### Sample Size

There were no previous trials of VRT in diabetics, hence no data to guide the likely target difference in renal function to be observed for the purpose of sample size calculation. No published data existed for the primary outcome of “fit for discharge” (chosen to minimize biases that can affect standard data on postoperative length of stay) and the sample size calculation described here uses information about actual postoperative length of stay as a proxy for the primary outcome. The median postoperative stay for diabetic patients having CABG (from our institutional database) is 7 days. Assuming time to “fit for discharge” is shorter than actual stay, we have proposed that the trial should be able to detect a 25% difference in the proportion of patients “fit for discharge” at 6 days between VRT and usual care groups (ie, 75% vs 50%). We have proposed that 170 participants would be required to detect this target difference with 90% power and 5% significance (2-tailed).

#### Statistical Analyses

Time to “fit for discharge” and length of Intensive Care Unit and postoperative hospital stays will be analyzed as time-to-event data using regression modelling for survival data. Means for continuous outcomes (transformed logarithmically if required) will be compared using regression modelling, adjusting for baseline covariates where available; “mixed models” will be used for outcomes with repeated measures such as eGFR and markers of glomerular, tubular, renal function, microRNA, CRP, and troponin T. Findings will be reported as effect sizes with 95% confidence intervals. The frequencies of complications and conversions will be tabulated descriptively. Analyses will be based on the intention-to-treat (as treated compared to intention-to-treat); conversions are expected to be rare.

A subgroup analysis comparing trial-specific (eg, UK or India) primary and secondary biochemical marker outcomes is prespecified in the statistical analysis plan. Subgroups will be compared by adding an allocation by trial interaction term into the model.

### Trial Status

Of the 491 patients assessed for eligibility during the study period, 169 patients (120 in UK, 49 in India) were successfully recruited and randomized over a 48-month period. Study recruitment was closed in July 2014. Study results are expected to be published in the summer of 2017.

## Discussion

### Principal Findings

The VeRDiCT study offers a unique opportunity to answer a fundamental question about a clinical intervention, which could reduce postoperative complications in an increasing proportion of atherosclerotic diabetic patients. To the best of our knowledge, at the time of study design there were no other ongoing trials investigating the clinical benefit of VRT in diabetic patients undergoing coronary surgery. To investigate the potential clinical efficacy of VRT, it is necessary to undertake a well-designed RCT to assess the impact of VRT on renal function and health outcomes in the selected patient cohort. The proposed research should contribute significantly to the understanding of the role of VRT in reducing postoperative complications in diabetic patients undergoing surgery and, if successful, contribute significantly to improving health care while reducing the burden on hospital resources. A multi-dimensional methodological approach based on the evaluation of an extensive list of objective and serial clinical, biochemical, and functional measures should prove valuable in characterizing the effects of VRT. Studying serial biochemical markers of renal and cardiac injury (and of inflammatory activation) at baseline, during surgery, and postoperatively might provide evidence for an organ-specific impact of VRT, which in turn should translate into health outcome benefits. In particular, a reduction in renal failure should affect the primary outcome of time to “fitness for discharge”. The methods used in this trial to assess renal, myocardial, and inflammatory function and activation have traditionally been employed in cardiac surgery and in diabetic patients [2-5,9].

Following discussion during the study design period, it was decided that the trial was not going to be blinded, as it was not practically possible to mask the infusion of the Hartmann’s solution. A covered drip could have been set up for all trial patients but it would have still been obvious to the patient and those responsible for their care whether or not a fluid infusion was being given. In addition, the prescription would have been visible in the medical record. However, the outcome endpoints of the trial are based on objective measures, hence the influence of the lack of blinding is minimal.

In undertaking this trial, the study team have encountered a number of logistical problems that reflect the difficulty of conducting research within a routine setting. The first was the variability of the information supplied to our institution (a tertiary referral center for cardiac surgery) upon referral of the patient. This variable made it difficult to establish which patients were diabetic, and this delay reduced the time available to approach the patient about the study, and for the patient to consider their participation. This issue was particularly problematic for urgent inpatients transferred directly from another hospital, often with very short notice. In these latter cases, this problem also had an impact on delivery of the intervention. The study was further hampered by the introduction of a *Day of Surgery Admission* (DOSA) policy implemented during the recruitment phase. The DOSA policy imposed a system by which elective patients would be preadmitted for a few hours for baseline evaluations two weeks before surgery, and were no longer admitted the evening before surgery, but on the morning of surgery. Patients admitted as DOSA could not be given the intervention, as there was not enough time to administer the 12-hour infusion of VRT. The study team was able to work with the waiting list coordinators to admit potential trial patients the night before surgery, but pressure on beds meant that this was not always possible.

The trial was conceived in Bristol, UK. The study design and protocol were discussed and agreed between the two trial teams following a preparatory visit to Kolkata by the UK CI. To enhance consistency across the trials, the Indian trial used the same protocol, study documents, and CRFs as the UK team. The Indian trial also used Sealed Envelope Ltd for randomization.

During the recruitment phase, there were two amendments to the study protocol. The first one consisted of removing the fixed 12-hour cap that allowed the patient to consider entering the trial; this was done to allow the inclusion of urgent inpatients referred at short notice from other hospitals. Inclusion of these patients is important to ensure that study results are generalizable to the study population that is likely to benefit from the intervention. Patients were only consented if they felt that they had had enough time to consider their participation. A second amendment (for a sub-cohort of patients) consisted of expanding the list of outcome measures, including a measure of baseline fasting glucose, an extra time point for sampling of blood and urine, and inclusion of microRNA, CRP, and troponin I as extra biochemical markers.

### Limitations

This is the first trial assessing the efficacy of preoperative VRT in diabetic patients undergoing coronary surgery on postoperative renal function and health outcomes. There are, however, some potential limitations: (1) only one dose of VRT was administered; (2) only the elective and urgent patient population was studied; and (3) logistical difficulties, such as the introduction of the DOSA policy, meant that patients otherwise eligible were not included. Future investigations should seek to determine the effects of higher doses of VRT, and doses that are administered during the postoperative period in these patients.

## Abbreviations

AKI: acute kidney injury
ASEPSIS: additional treatment, serous discharge, erythema, purulent exudate, separation of the deep tissues, isolation of bacteria, and the duration of inpatient stay
BHF: British Heart Foundation
CABG: coronary artery bypass graft
CI: chief investigator
CRF: case report form
CROQ: Coronary Revascularization Outcome Questionnaire
CRP: C-reactive protein
DM: diabetes mellitus
DOSA: Day of Surgery Admission
eGFR: estimated glomerular filtration rate
GFR: glomerular filtration rate
GWT: Garfield Weston Trust
IMP: Investigational Medicinal Product
MHRA: Medicines and Healthcare Products Regulatory Agency
NHS: National Health Service
NIHR: National Institute for Health Research
PIS: Patient Information Sheet
RCT: randomized controlled trial
RIFLE: Risk, Injury, Failure, Loss, and End stage
RNA: ribonucleic acid
RTIICS: Rabindranath Tagore International Institute of Cardiac Sciences
REC: Research Ethics Committee
UK: United Kingdom
VeRDiCT: Volume Replacement in Diabetic Patients Undergoing Coronary Artery Bypass Grafting Surgery: Protocol for a Randomized Controlled Trial
VRT: Volume Replacement Therapy

## References

[1] Beckman JA, Creager MA, Libby P (2002). Diabetes and atherosclerosis: epidemiology, pathophysiology, and management. JAMA.

[2] Thakar CV, Christianson A, Himmelfarb J, Leonard AC (2011). Acute kidney injury episodes and chronic kidney disease risk in diabetes mellitus. Clin J Am Soc Nephrol.

[3] Adler DS, Goldman L, O'Neil A, Cook EF, Mudge GH, Shemin RJ, DiSesa V, Cohn LH, Collins JJ (1986). Long-term survival of more than 2,000 patients after coronary artery bypass grafting. Am J Cardiol.

[4] Thourani VH, Weintraub WS, Stein B, Gebhart SS, Craver JM, Jones EL, Guyton RA (1999). Influence of diabetes mellitus on early and late outcome after coronary artery bypass grafting. Ann Thorac Surg.

[5] Rajakaruna C, Rogers C, Suranimala C, Angelini G, Ascione R (2006). The effect of diabetes mellitus on patients undergoing coronary surgery: a risk-adjusted analysis. J Thorac Cardiovasc Surg.

[6] Ascione R, Rogers CA, Rajakaruna C, Angelini GD (2008). Inadequate blood glucose control is associated with in-hospital mortality and morbidity in diabetic and nondiabetic patients undergoing cardiac surgery. Circulation.

[7] Kubal C, Srinivasan AK, Grayson AD, Fabri BM, Chalmers JA (2005). Effect of risk-adjusted diabetes on mortality and morbidity after coronary artery bypass surgery. Ann Thorac Surg.

[8] Bahar I, Akgul A, Ozatik MA, Vural KM, Demirbag AE, Boran M, Tasdemir O (2005). Acute renal failure following open heart surgery: risk factors and prognosis. Perfusion.

[9] Harty J (2014). Prevention and management of acute kidney injury. Ulster Med J.

[10] Zakkar M, Bruno VD, Guida G, Angelini GD, Chivasso P, Suleiman MS, Bryan AJ, Ascione R (2016). Postoperative acute kidney injury defined by RIFLE criteria predicts early health outcome and long-term survival in patients undergoing redo coronary artery bypass graft surgery. J Thorac Cardiovasc Surg.

[11] Hawkes N (2013). Acute kidney injury is a more important safety issue than MRSA, says NICE. BMJ.

[12] National Institute for Health and Care Excellence (2013). NICE Clinical Guideline CG169.

[13] Thakar CV, Worley S, Arrigain S, Yared J, Paganini EP (2005). Influence of renal dysfunction on mortality after cardiac surgery: modifying effect of preoperative renal function. Kidney Int.

[14] Magee MJ, Dewey TM, Acuff T, Edgerton JR, Hebeler JF, Prince SL, Mack MJ (2001). Influence of diabetes on mortality and morbidity: off-pump coronary artery bypass grafting versus coronary artery bypass grafting with cardiopulmonary bypass. Ann Thorac Surg.

[15] Borthwick E, Ferguson A (2010). Perioperative acute kidney injury: risk factors, recognition, management, and outcomes. BMJ.

[16] Epstein BJ (2004). Elevations in serum creatinine concentration: concerning or reassuring?. Pharmacotherapy.

[17] Juhlin T, Björkman S, Gunnarsson B, Fyge A, Roth B, Höglund P (2004). Acute administration of diclofenac, but possibly not long term low dose aspirin, causes detrimental renal effects in heart failure patients treated with ACE-inhibitors. Eur J Heart Fail.

[18] Schoolwerth AC, Sica DA, Ballermann BJ, Wilcox CS, Council on the Kidney in Cardiovascular Diseasethe Council for High Blood Pressure Research of the American Heart Association (2001). Renal considerations in angiotensin converting enzyme inhibitor therapy: a statement for healthcare professionals from the Council on the Kidney in Cardiovascular Disease and the Council for High Blood Pressure Research of the American Heart Association. Circulation.

[19] Grocott MP, Mythen MG, Gan TJ (2005). Perioperative fluid management and clinical outcomes in adults. Anesth Analg.

[20] Schnuelle P, Johannes van der Woude F (2006). Perioperative fluid management in renal transplantation: a narrative review of the literature. Transplant Int.

[21] Kreimeier U, Peter K (1998). Strategies of volume therapy in sepsis and systemic inflammatory response syndrome. Kidney Int Suppl.

[22] Angelini GD, Taylor FC, Reeves BC, Ascione R (2002). Early and midterm outcome after off-pump and on-pump surgery in Beating Heart Against Cardioplegic Arrest Studies (BHACAS 1 and 2): a pooled analysis of two randomised controlled trials. The Lancet.

